# Regulation of Centromere Localization of the *Drosophila* Shugoshin MEI-S332 and Sister-Chromatid Cohesion in Meiosis

**DOI:** 10.1534/g3.114.012823

**Published:** 2014-07-31

**Authors:** Cristina Nogueira, Helena Kashevsky, Belinda Pinto, Astrid Clarke, Terry L. Orr-Weaver

**Affiliations:** * Whitehead Institute, Massachusetts Institute of Technology, Cambridge, Massachusetts 02142; † Department of Biology, Massachusetts Institute of Technology, Cambridge, Massachusetts 02142

**Keywords:** chromosome segregation, chromosome nondisjunction, precocious sister-chromatid separation, chromosome passenger complex

## Abstract

The Shugoshin (Sgo) protein family helps to ensure proper chromosome segregation by protecting cohesion at the centromere by preventing cleavage of the cohesin complex. Some Sgo proteins also influence other aspects of kinetochore-microtubule attachments. Although many Sgo members require Aurora B kinase to localize to the centromere, factors controlling delocalization are poorly understood and diverse. Moreover, it is not clear how Sgo function is inactivated and whether this is distinct from delocalization. We investigated these questions in *Drosophila melanogaster*, an organism with superb chromosome cytology to monitor Sgo localization and quantitative assays to test its function in sister-chromatid segregation in meiosis. Previous research showed that in mitosis in cell culture, phosphorylation of the Drosophila Sgo, MEI-S332, by Aurora B promotes centromere localization, whereas Polo phosphorylation promotes delocalization. These studies also suggested that MEI-S332 can be inactivated independently of delocalization, a conclusion supported here by localization and function studies in meiosis. Phosphoresistant and phosphomimetic mutants for the Aurora B and Polo phosphorylation sites were examined for effects on MEI-S332 localization and chromosome segregation in meiosis. Strikingly, MEI-S332 with a phosphomimetic mutation in the Aurora B phosphorylation site prematurely dissociates from the centromeres in meiosis I. Despite the absence of MEI-S332 on meiosis II centromeres in male meiosis, sister chromatids segregate normally, demonstrating that detectable levels of this Sgo are not essential for chromosome congression, kinetochore biorientation, or spindle assembly.

Accurate chromosome segregation is essential to prevent aneuploidy; in mitosis, such alterations in chromosome number are associated with cancer and tumor progression and in meiosis with miscarriage and birth defects (Siegel and Amon 2012; Duijf and Benezra 2013; Jones and Lane 2013; Ricke and van Deursen 2013). Sister-chromatid cohesion, a physical link between replicated chromatids, is essential to establish stable bipolar kinetochore microtubule attachments and thus proper chromosome segregation. The cohesin complex is required for cohesion, and cleavage of the Scc1/Rad21 (Rec8 in meiosis) subunit by separase at the metaphase/anaphase transition is sufficient for release of cohesion and movement of sister chromatids to opposite poles (Oliveira and Nasmyth 2010). In metazoan mitosis, cohesin along the chromosome arms is released during prophase by a mechanism independent of cleavage, whereas the pool at the centromere is protected until the metaphase/anaphase transition. In meiosis I, cohesin on the arms is cleaved as homologs segregate but is protected from cleavage at the centromere. Thus, a link is retained between sister chromatids after metaphase I and this cohesion ensures proper sister segregation in meiosis II.

Maintenance of cohesion at the centromere is mediated by distinct members of the Shugoshin (Sgo) family of proteins that protect cohesin at the centromere in mitosis and meiosis, at least in part by recruiting the PP2A-B′ phosphatase to dephosphorylate cohesin subunits, rendering them insensitive to cleavage (Clift and Marston 2011; Gutierrez-Caballero *et al.* 2012). For example, mammalian Sgol1 protects centromere cohesin from the mitotic prophase removal pathway, whereas Sgol2 acts in meiosis (McGuinness *et al.* 2005; Llano *et al.* 2008). Sgo proteins have been reported to have additional roles such as control of chromosome congression by recruiting Mitotic Centromere-Associated Kinase (MCAK) to the kinetochore, sensing of tension at the centromere, promotion of kinetochore biorientation in mitosis, stabilizing cohesin on chromosome arms, spindle assembly, inactivation of the Spindle Assembly Checkpoint, and control of centriole cohesion (Rivera and Losada 2009; Tanno *et al.* 2010; Clift and Marston 2011; Rivera *et al.* 2012; Rattani *et al.* 2013; Verzijlbergen *et al.* 2014).

The founding member of the Sgo family was identified by mutations in the *Drosophila mei-S332* gene that cause loss of centromere cohesion beginning in anaphase I, resulting in random segregation of sister chromatids in meiosis II (Davis 1971; Goldstein 1980; Kerrebrock *et al.* 1992). Although cohesion is lost prematurely in meiosis I, it is in anaphase I, after the homologs have segregated. Consequently, the separated defects are not manifest until meiosis II, when separated sister chromatids fail to establish stable bipolar microtubule attachments to ensure accurate segregation. When cloned, the protein was shown to localize to centromeres from prophase I until the metaphase II/anaphase II transition (Kerrebrock *et al.* 1995). It has been shown to affect cohesin, being necessary to maintain centromere localization of this complex in meiosis in males (Yan *et al.* 2010). MEI-S332 is the sole Sgo identified in *Drosophila*. Although not essential for centromere cohesion in mitosis, MEI-S332 localizes to mitotic centromeres and contributes to centromere cohesion in mitosis (Moore *et al.* 1998; Leblanc *et al.* 1999; Lee *et al.* 2005).

Several studies have identified regulatory steps for Sgo localization and delocalization at the centromere. MEI-S332 binds to the inner centromere protein (INCENP) of the chromosome passenger complex (CPC) and is phosphorylated by the CPC Aurora B kinase subunit (Resnick *et al.* 2006). Mutation of these phosphorylation sites reduces centromere localization of MEI-S332 in mitotic cell culture, and there also is loss of specific centromere localization in meiosis in *incenp* mutants (Resnick *et al.* 2006). Polo kinase mutants lead to persistence of MEI-S332 on the centromere in anaphase of mitosis and anaphase II of meiosis (Clarke *et al.* 2005). This may be a direct effect of Polo phosphorylation, as Polo binds MEI-S332 via a Polo Binding Domain (PBD). Mutation of the priming site in the PBD of MEI-S332 blocks delocalization in anaphase in cell culture (Clarke *et al.* 2005). In fission yeast, Sgo localization requires phosphorylation of histone H2A by Bub1 kinase, and Sgo and the CPC reciprocally promote each other’s localization to the centromere (Kawashima *et al.* 2010; Tanno *et al.* 2010). A similar relationship between the CPC and Sgo exists in *Xenopus* extracts, human cells, and with Sgol2 in mouse meiosis (Tsukahara *et al.* 2010; Yamagishi *et al.* 2010; Rivera *et al.* 2012; Rattani *et al.* 2013).

It remains to be determined how protection of cohesin by Sgo proteins is inactivated to permit cohesin cleavage and release of cohesion. Delocalization from the centromere could be necessary and sufficient to release cohesion, or there could be a mechanism to inactivate Sgo proteins independently of localization. A recent hypothesis is that the tension resulting from stable bipolar attachment of microtubules to kinetochores pulls the Sgo proteins toward the kinetochore, away from the centromere (Gomez *et al.* 2007; Lee *et al.* 2008; Clift and Marston 2011). This may dissociate it from cohesin and/or the PP2A-B’ phosphatase.

We have exploited mutated forms of MEI-S332 that affect centromere localization in meiosis to examine the relationship between localization and control of centromere cohesion in meiosis. These studies support the suggestion from previous studies in cell culture that cohesion can be released without centromere delocalization of MEI-S332, and they indicate that MEI-S332 is not required at the centromere after anaphase I.

## Material and Methods

### Generation of MEI-S332 Aurora B phosphorylation mutants and Polo binding-site mutants

The previously described wild-type *mei-S33*2-*GFP* fusion on a genomic construct (Kerrebrock *et al.* 1995) was used as a template for the generation of the MEI-S332^S124-126A or D^ phosphorylation site mutants and the MEI-S332^T331A or D^ mutant constructs. A *Swa*I/*Xho*I fragment of wild-type *mei-S332* was cloned into *Sma*I/*Xho*I sites in BSSK+ and mutations were generated using Phusion Site-Directed Mutagenesis Kit according to manufacturer’s instructions. The mutations were confirmed by sequencing and swapped into the original wild-type *mei-S332* construct using either *Pac*I/*Bst*EI restriction enzymes (in the case of S124-126A or D mutations) or *Bst*EI/*Sph*I enzymes (in the case of T331A or D mutations). Transformation was carried out by the standard wings-clipped *P* element transposase approach. Insertions on the third chromosome were selected, and these transformants were then crossed to *yw/^y+^Y*; *mei-S332^4^/ SM6* or *yw/^y+^Y*; *mei-S332^7^ /SM6* to get *yw/^y+^Y*; *mei-S332^4 or 7^/ SM6*; *[mei-S332^S124-126A or D^]* or *[mei-S332^T331A or D^]* males or *yw/yw*; *mei-S332^4 or 7^/SM6*; *[mei-S332^S124-126A or D^]* or *[mei-S332^T331A or D^]* females.

### Nondisjunction tests

Nondisjunction tests were performed as described (Kerrebrock *et al.* 1992). For analysis of male nondisjunction *yw/^y+^Y*; *mei-S332^4^/ mei-S332^7^*; *[mei-S332^S124-126A or D^] or [mei-S332^T331A or D^]* were crossed to attached-*X*, *y^2^ su(w^a^) w^a^* virgin females. Controls were the mutant *yw/^y+^Y*; *mei-S332^4^/ mei-S332^7^* and the mutants with the wild-type *mei-S332-GFP* transgene. For analysis of nondisjunction in females *yw/yw*; *mei-S332^4^/ mei-S332 ^7^*; *[mei-S332^S124-126A or D^] or [mei-S332^T331A or D^]* females were crossed to attached *X-Y*, *v f B* males. Crosses were set up at 25° with 15 virgin females per bottle. Parents were flipped to a new bottle after 5 days and discarded in 5 more days. Progeny were scored for 18 days from day 0. To determine whether nondisjunction frequencies were different the Mann-Whitney *U*-test and Wilcoxon two sample test for two samples (ranked observations, not paired) were used, and a probability less that 0.05 was scored as significant.

### Testes squashes and spermatocyte immunofluorescence

Testes from young adult *yw/^y+^Y*; *mei-S332^4^/ mei-S332^7^*; *[mei-S332^S124-126A or D^ ]* or [*mei-S332^T331A or D^*] male flies were prepared as described (Bonaccorsi *et al.* 2000). Immunostaining of spermatocytes was performed as follows: squashes were frozen in liquid nitrogen and then transferred to 95% ethanol for at least 10 min. Slides were fixed in 4% formaldehyde (252549; Sigma-Aldrich, washed in PBT (phosphate-buffered saline + 0.1% Triton X-100) and blocked for 30 min in 3% bovine serum albumin/PBT. Slides were then incubated with primary antibodies (guinea pig anti-MEI-S332 at 1:5,000 and mouse anti-tubulin DM1A at 1:500) overnight at 4°. Secondary antibodies were used at 1:500 (RRX or Alexa Fluor 488-conjugated antiguinea pig and FITC or LRSC-conjugated antimouse; The Jackson Laboratory). Slides were stained with DAPI (4′,6-diamidino-2-phenylindole dihydrochloride) in phosphate-buffered saline at 0.4 μg/mL and mounted in Vectashield (Vector Laboratories).

### Microscopy

Spermatocyte images were collected using either a Nikon eclipse Ti microscope with a 60X oil objective and a Hamamatsu (model c4742-80-12AG) camera with NIS-Elements Microscope Imaging Software, or on a Zeiss Axio Imager.Z1 with a 60X oil objective using an AxioCam MRm Rev.3 camera and Axiovision 4.8 release software.

## Results

### Dynamic localization of MEI-S332 between meiosis I and II

We previously found that MEI-S332 is present on centromeres in spermatocytes from prophase I until late anaphase I and in meiosis II, but we had not examined its distribution between the meiotic divisions when chromosomes decondense (Kerrebrock *et al.* 1995). Using antibodies to MEI-S332, we analyzed its localization in meiosis in spermatocytes. Because spermatocytes undergo meiosis without any developmental arrests, it is possible to recover all stages of meiosis, including interphase between the two divisions. We confirmed that the antibody specifically recognizes the MEI-S332 protein by staining testes from *mei-S332^4^/mei-S332^7^* transheterozygous males, which lack detectable protein (Supporting Information, Figure S1).

**Figure 1:**
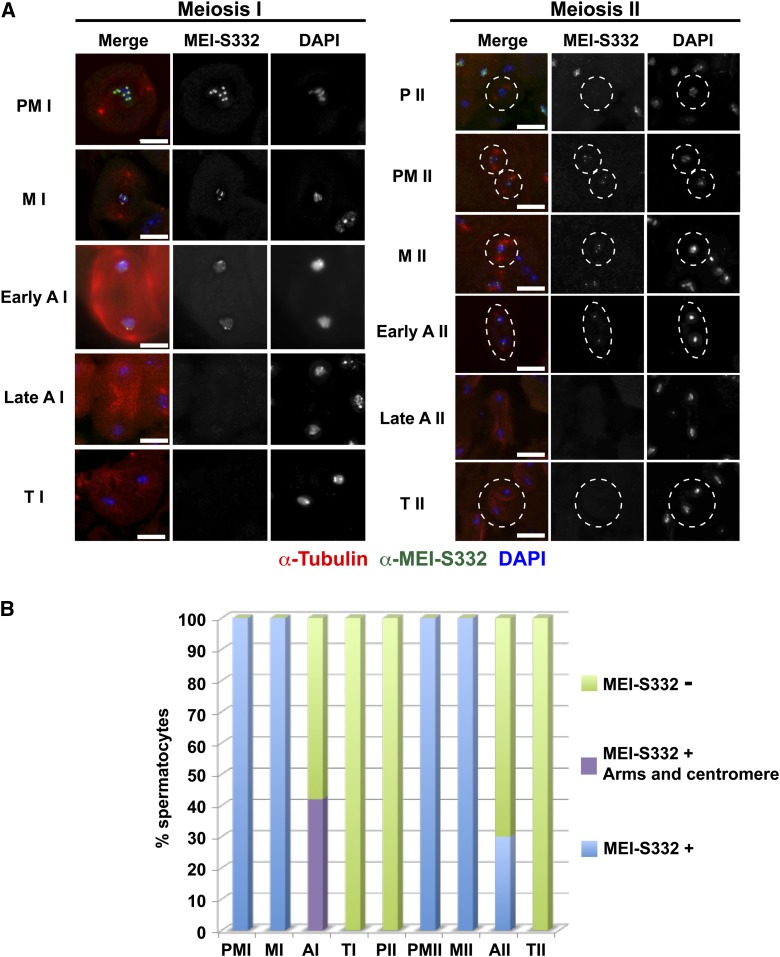
Dynamic distribution of MEI-S332 in male meiosis. (A) Localization of MEI-S332 through meiosis. Meiotic stages are labeled on the left: PM I, prometaphase I; M I, metaphase I; A I, anaphase I (early or late); T I, telophase I; P II, prophase II; PM II, prometaphase II; M II, metaphase II; A II, anaphase II (early or late); T II, telophase II. Merged panels show MEI-S332 antibody staining in green, tubulin in red, and DAPI in blue. Split channels are shown for MEI-S332 and DAPI. The dotted circles for the meiosis II panels demarcate individual spermatocytes. Scale bars = 10 μm. During metaphase I, MEI-S332 is detected enriched at the centromeres of the condensed chromosomes. By anaphase I, MEI-S332 is still found at centromeric dots but delocalizes from centromeres and becomes spread over the chromosome arms. Early and late anaphase I were distinguished by spindle morphology and the appearance of the spindle midzone. MEI-S332 is no longer detectable on chromosomes in late anaphase I. MEI-S332 is not present on chromosomes in telophase I and prophase II. In prometaphase II, MEI-S332 is detectable again at centromeres, persisting until early anaphase II. (B) Quantification of MEI-S332 localization. Blue indicates MEI-S332 observed solely in centromere foci, purple MEI-S332 detectable along the chromosomes and at the centromeres, and green no MEI-S332 visualized on the chromosomes. Numbers scored were: prometaphase I (n = 48), metaphase I (n = 28), anaphase I (n = 12), telophase I (n = 39), prophase II (n = 5), prometaphase II (n = 16), metaphase II (n = 18), anaphase II (n = 29), and telophase II (n = 65). The anaphase I bar includes both early and late anaphase I cells.

Two unexpected observations arose from anti-MEI-S332 staining of wild-type testes. First, although MEI-S332 is enriched at the centromere in early anaphase I, it is also detectable at lower levels throughout the chromosomes, revealing a redistribution at the metaphase I/anaphase I transition (Figure 1). Enhanced sensitivity of the anti-MEI-S332 antibody compared to the GFP signal used in previous studies likely accounts for our ability to observe the protein along the chromosome arms. The protein is not detectable on chromosomes in telophase I and prophase II but is localized to the centromeres in prometaphase II. The second new observation afforded by the antibody staining is that the protein is still present in the centromere region in some anaphase II cells. We do not observe it along chromosome arms as in early anaphase I cells. Because the protein does not delocalize completely at the metaphase II/anaphase II transition, there appears to be a mechanism to release cohesion without complete delocalization of MEI-S332, consistent with the cell culture studies.

This redistribution of MEI-S332 in late meiosis I followed by apparent re-recruitment to the centromere between prophase II and metaphase II is similar to the localization properties described for the CPC in *Drosophila* spermatocytes, although the CPC remains detectable on the chromosome arms in anaphase II (Resnick *et al.* 2006). In contrast, in mouse meiosis Sgol2 is retained on the centromeres in telophase I, but it also is not present in interkinesis (Gomez *et al.* 2007; Parra *et al.* 2009).

### Localization and function of MEI-S332 protein mutated for Aurora B phosphorylation sites

MEI-S332 centromere localization requires the CPC, and mutations in the *incenp* subunit resulted in MEI-S332 being spread along the chromosomes in meiosis I (Resnick *et al.* 2006). In mitosis in cultured cells, knock down of Aurora B similarly caused MEI-S332 to be dispersed on the chromosomes rather than specifically localized to the centromeres in metaphase. MEI-S332 binds directly to INCENP and is phosphorylated *in vitro* by Aurora B. Mutation of serines 124, 125, and 126 to alanines inhibited phosphorylation by Aurora B *in vitro*, and when expressed in cell culture localization of this mutant form of MEI-S332 to the centromere was reduced (Resnick *et al.* 2006).

Given the dependency of MEI-S332 localization to be restricted to the centromere and the reduced localization of the Aurora B phosphomutant form, we wanted to test the localization and functional properties of this mutant form of MEI-S332 *in vivo* in meiosis. To this end, we made transgenic lines in which MEI-S332 with these three serine-alanine substitutions (S124,5,6-A) was expressed under the normal promoter. The protein contained a GFP fusion to quantify expression levels relative to the endogenous protein by immunoblot (Figure S2).

**Figure 2:**
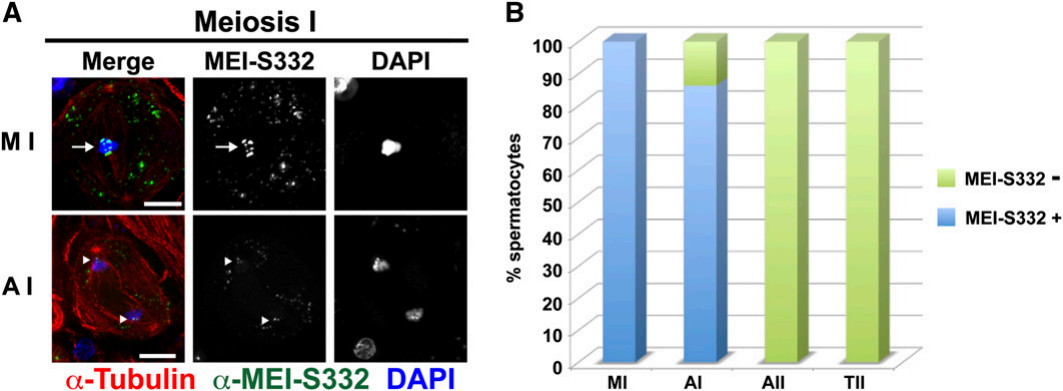
Localization of a MEI-S332 mutant for the Aurora B phosphorylation sites (MEI-S332^S124,5,6-A^) during male meiosis. (A) Localization of MEI-S332^S124,5,6-A^ in metaphase I and early anaphase I. Labels, colors, and scale bars as in Figure 1. The arrow points to the metaphase I plate. Arrowheads in the early Anaphase I panel show MEI-S332^S124,5,6-A^ present at the centromeres. In contrast to the wild-type protein, we did not observe MEI-S332^S124,5,6-A^ dispersed along chromosome arms in anaphase I or present on anaphase II chromosomes, although given the low number of anaphase II cells, we may have failed to detect it. Note that as observed previously, MEI-S332-GFP protein is observed in puncta in the cytoplasm of primary spermatocytes (Kerrebrock *et al.* 1995). (B) Quantification of MEI-S332^S124,5,6-A^ localization in metaphase I (n = 44), anaphase I (n = 51), anaphase II (n = 5), and telophase II (n = 251). Metaphase II figures were not observed in these spermatocytes, possibly because of a faster onset of anaphase II. Cells were scored as having MEI-S332 if any signal was detectable. The levels of protein at the centromere in anaphase I were reduced relative to wild type.

The mutant form of MEI-S332 resistant to Aurora B phosphorylation *in vitro* was localized in spermatocytes lacking endogenous MEI-S332 by staining with the MEI-S332 antibody (Figure 2). In contrast to the studies in mitotic cell culture, MEI-S332^S124,5,6-A^ localizes to metaphase I centromeres in spermatocytes at frequencies comparable to wild type. This actual protein localization appears weakened relative to wild type, however. Even though the protein could be observed on centromeres in some early anaphase I cells, the staining intensity was reduced compared to wild type. In other primary spermatocytes it was not detectable along the arms or even at the centromeres in early anaphase I (Figure 2B). The Aurora B phosphoresistant form of MEI-S332 is absent from chromosomes in anaphase II (although the number recovered was low) and telophase II (Figure 2B). We did not recover metaphase II cells expressing this form of MEI-S332, perhaps because the metaphase II/anaphase II transition is accelerated. These results are consistent with interaction between MEI-S332 and CPC contributing to centromere localization of MEI-S332 in meiosis as well as mitosis.

We used nondisjunction tests with marked sex chromosomes to quantify the functionality of the Aurora B phosphoresistant form of MEI-S332 in protecting sister-chromatid cohesion at the centromere in meiosis I. In *mei-S332* null mutants, loss of sister-chromatid cohesion results in sperm lacking sex chromosomes or containing two sister *X* chromosomes (Table 1). Sperm with an *X* and *Y* chromosome, indicative of meiosis I nondisjunction, occur at low frequencies. A transgene expressing a wild-type MEI-S332-GFP fusion protein rescues these meiotic missegregation events (Table 1). The transgene lines in which the MEI-S332^S124,5,6-A^ mutants are expressed also restore proper meiotic segregation to *mei-S332* null mutants. A line that fails to express the fusion protein does not rescue (Figure S2 and Table 1). Only this line (7a) shows a statistically significant difference in percent exceptional progeny from rescue with the wild-type MEI-S332-GFP fusion. Thus, although localization of centromeric MEI-S332 is weakened relative to wild type, centromere cohesion of sister chromatids is adequately protected by the MEI-S332^S124,5,6-A^ protein to ensure accurate chromosome segregation.

**Table 1:** Sex chromosome nondisjunction in males transheterozygous for *mei-S332* null alleles with transgenic *mei-S332^S124,5,6^* phosphomutants

	Regular Sperm		Exceptional Sperm				Total Progeny	Total Exceptional Progeny (%)
	*Y (Y)**^a^*	*X*	Nullo-*XY* (%)	*XX* (%)	*XY(Y)* (%)	*XXY(Y)* (%)		
*yw/^y+^Y;Tft/SM6 control*	372	566	0	0	0	0	938	0 (0%)
*mei-S332^7^ /mei-S332^4^*	224	278	59 (10%)	25 (4.2%)	3 (0.5%)	0 (0%)	589	87 (14.77%)
*mei-S332^7^ /mei-S332^4^*; *P{w^+^*, *mei-S332^+^-GFP}*	947	1161	11 (0.52%)	0 (0%)	3 (0.14%)	0 (0%)	2122	14 (0.66%)
*mei-S332^7^ /mei-S332^4^*; *P{w^+^*, *mei-S332^S124,5,6-A^-GFP} line 3b*	534	676	8 (0.65%)	1 (0.08%)	0 (0%)	0 (0%)	1219	9 (0.74%)
*mei-S332^7^ /mei-S332^4^*; *P{w^+^*, *mei-S332^S124,5,6-A^-GFP} line 5a*	600	742	6 (0.44%)	0 (0%)	1 (0.074%)	0 (0%)	1349	7 (0.52%)
*mei-S332^7^ /mei-S332^4^*; *P{w^+^*, *mei-S332^S124,5,6-A^-GFP} line7a**^b^*	39	49	19 (16.7%)	7 (6.14%)	0 (0%)	0 (0%)	114	26 (22.81%)
*mei-S332^7^ /mei-S332^4^*; *P{w^+^*, *mei-S332^S124,5,6-D^-GFP} line 4a*	440	543	4 (0.40%)	0 (0%)	2 (0.20%)	0 (0%)	989	6 (0.61%)
*mei-S332^7^ /mei-S332^4^*; *P{w^+^*, *mei-S332^S124,5,6-D^-GFP} line 6a*	269	323	2 (0.33%)	0 (0%)	2 (0.33%)	0 (0%)	596	4 (0.67%)
*mei-S332^7^ /mei-S332^4^*; *P{w^+^*, *mei-S332^S124,5,6-D^-GFP} line 13a*	600	789	13 (0.92%)	0 (0%)	6 (0.43%)	0 (0%)	1408	19 (1.35%)
*mei-S332^7^ /mei-S332^4^*; *P{w^+^*, *mei-S332^S124,5,6-D^-GFP} line17c*	383	509	1 (0.11%)	0 (0%)	1 (0.11%)	0 (0%)	894	2 (0.22%)

a Diplo-*Y* sperm cannot be distinguished from regular sperm with a single *Y* chromosome.

b This line shows no transgenic protein expression by immunoblot (see Figure S2).

### Localization of MEI-S332 after anaphase I is not required for proper chromosome segregation

**Figure 3:**
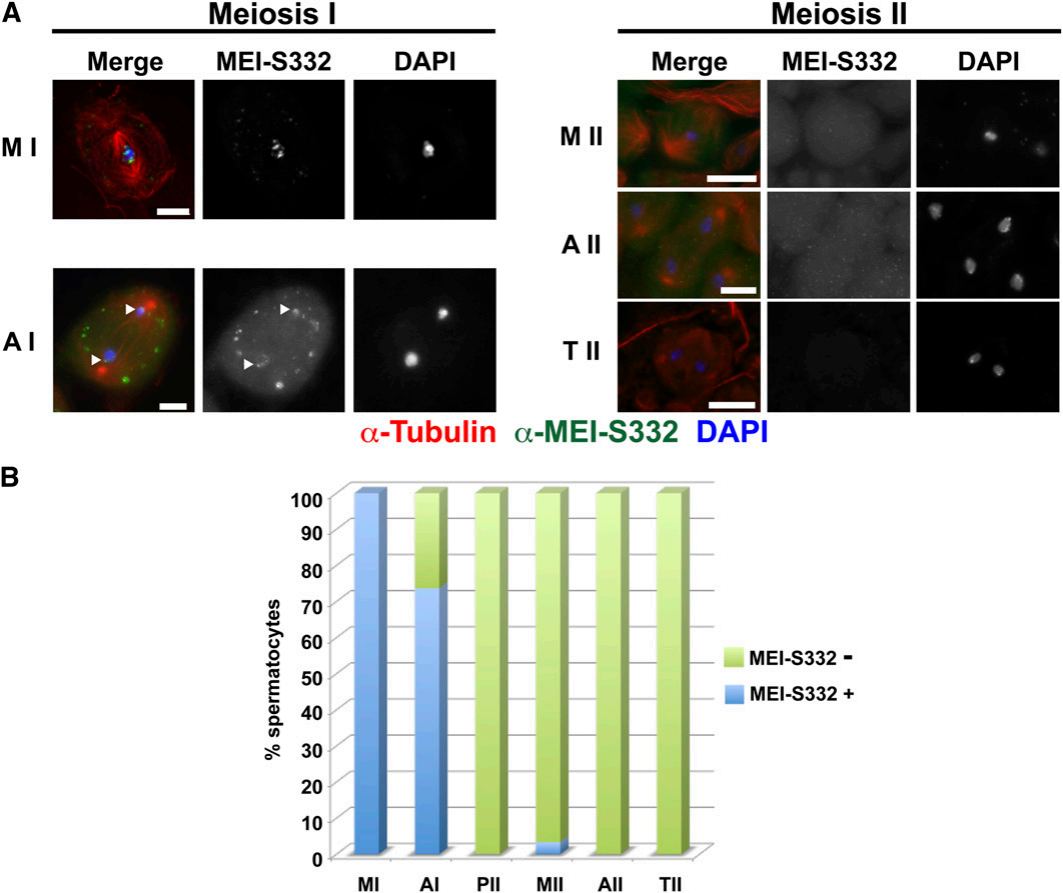
Localization of a MEI-S332 mimic for Aurora B phosphorylation sites (MEI-S332^S124,5,6-D^) during male meiosis. (A) Localization of MEI-S332^S124,5,6-D^ in meiosis I and II. Labels, colors, and scale bars as in Figure 1. Arrowheads in the early Anaphase I panel show MEI-S332^S124,5,6-D^ present at the centromeres. In contrast to the wild-type protein, we did not observe MEI-S332^S124,5,6-D^ dispersed along chromosome arms in early anaphase I, and the mutant protein remains unlocalized to chromosomes throughout meiosis II. Cytoplasmic foci of MEI-S332-GFP are present in the primary spermatocytes. (B) Quantification of MEI-S332^S124,5,6-D^ localization in metaphase I (n = 5), anaphase I (n = 19), metaphase II (n = 60), anaphase II (n = 15), and telophase II (n = 164).

Given that mutations in MEI-S332 that inhibit phosphorylation by Aurora B reduce centromere localization both in cell culture mitosis and in meiosis, we constructed mutations that cause phosphomimetic amino acid substitutions at the three serines required for Aurora B phosphorylation. Transgenic lines were made expressing the S124,5,6-D mutant MEI-S332-GFP at levels comparable with wild type (Figure S2). The mutant protein was localized in *mei-S332*−null mutants. Although the S124,5,6-D mutant protein localizes to the centromere in metaphase I, it was not detectable on the centromeres of 30% of anaphase I cells, was not present along chromosomes in early anaphase I, and was almost entirely absent from centromeres in meiosis II (Figure 3).

The absence of MEI-S332 in meiosis II permitted us to test whether MEI-S332 is required at the centromere or on the chromosomes during these stages of meiosis for accurate chromosome segregation. The cohesion protection function of MEI-S332 would not necessitate the presence of the protein in meiosis II. This is because cohesin needs to be protected from separase cleavage at the centromere at the metaphase I/anaphase I transition. If cohesin is not protected and cleaved, sister chromatids lose cohesion at this transition. Meiosis I segregation is not affected, because correct kinetochore-microtubule attachments have already been established. If separated in anaphase I, however, the sister chromatids lack a physical attachment to ensure proper kinetochore-microtubule attachment in meiosis II. Thus, a defect that occurred in meiosis I is read out as a segregation defect in meiosis II. But it is possible that MEI-S332 has additional segregation roles after the protection function at metaphase I/anaphase I. Sgo family members have been reported to be required for mitotic chromosome congression and kinetochore biorientation. If MEI-S332 were required for chromosome congression or kinetochore biorientation in meiosis II, mechanistically equivalent to mitosis, then nondisjunction would be predicted in strains in which the S124,5,6-D mutant is the sole form of MEI-S332 present.

We used genetic nondisjunction assays to evaluate the function of MEI-S332^S124,5,6-D^ in both meiotic divisions in male meiosis (Table 1). The mutant form of the protein restored meiotic chromosome segregation comparable to the wild-type MEI-S332-GFP transgene. Only one transgenic line, 13a, exhibited nondisjunction levels significantly different than the wild-type transgene, and this is likely a consequence of the mutant protein being expressed at lower levels than wild type (Table 1, Figure S2). Thus, despite the absence of detectable chromosomally localized protein in meiosis II, the chromosomes segregate accurately. This finding is consistent with MEI-S332 being required on chromosomes until anaphase I and lacking a role at the centromere in meiosis II in males.

### Analysis of meiosis in MEI-S332 mutants affecting Polo binding

We previously demonstrated that mutation of a putative priming threonine in a Polo box binding domain to alanine (T331-A) both decreased *in vitro* binding to Polo and Plx1-dependent phosphorylation of MEI-S332 in *Xenopus* anaphase extracts (Clarke *et al.* 2005). *In vivo*, we observed retention of MEI-S332 on mitotic anaphase and meiotic anaphase II centromeres in *polo* mutants, and we observed that *polo* mutants dominantly suppressed *mei-S332* mutants. The *in vitro* and *in vivo* results suggested that phosphorylation of MEI-S332 by Polo leads to its dissociation from the centromere at anaphase. Consistent with this finding, the T331-A mutant MEI-S332 protein did not dissociate from centromeres in anaphase when expressed in cell culture, and additionally it localized along the chromosomes in telophase (Clarke *et al.* 2005). Nevertheless, centromere cohesion was released, implying that MEI-S332 mediated protection of cohesion could be inactivated without dissociation of the protein from the centromere.

**Figure 4:**
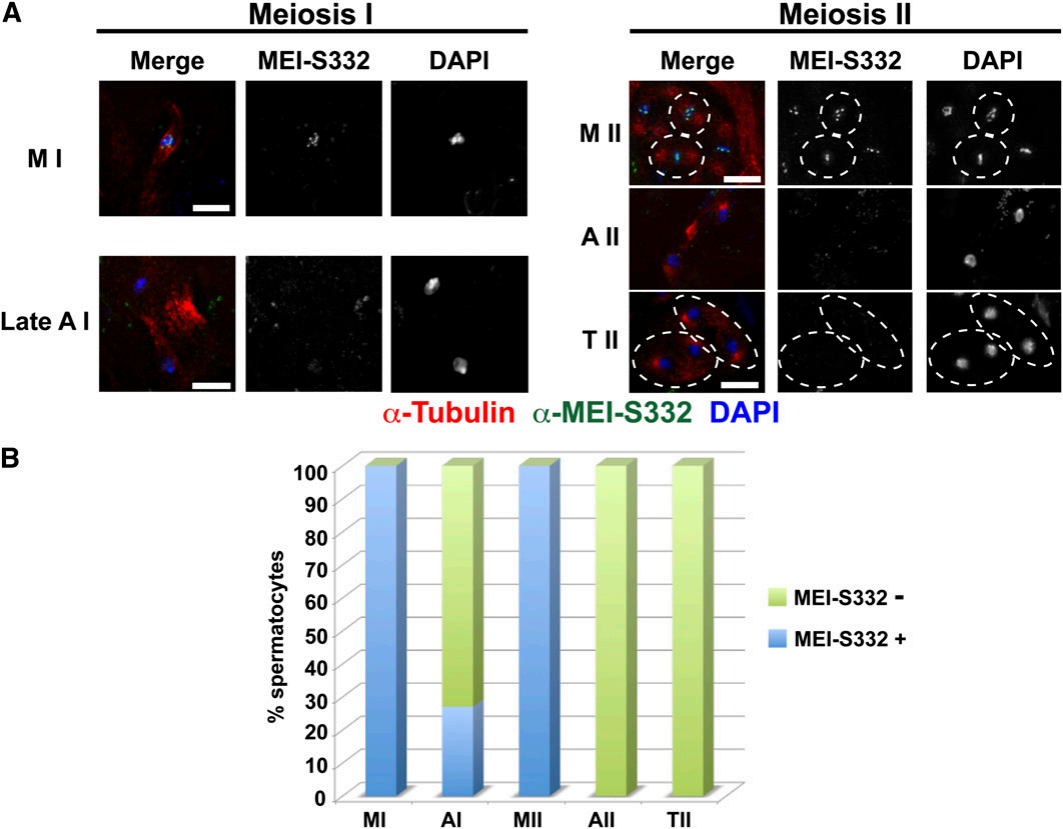
*In vivo* localization of the MEI-S332^T331-A^ mutant protein. (A) Localization of MEI-S332^T331-A^ in meiosis I and II. Labels, colors, and scale bars as in Figure 1. A late anaphase I spermatocyte is shown. The dotted lines in the meiosis II panels demarcate two metaphase II and two telophase II spermatocytes. (B) Quantification of MEI-S332^T331-A^ localization. Spermatocyte numbers scored were: metaphase I (n = 21), anaphase I (n = 22), metaphase II (n = 11), anaphase II (n = 17), and telophase II (n = 65). This mutant protein localizes and delocalizes from centromeres with proper timing in meiosis I spermatocytes, although in contrast to wild type, we did not observe the protein along chromosome arms in early anaphase I. We did not detect the protein localized on the anaphase II chromosomes.

To test the localization properties and function of the T331-A mutant in meiosis, we produced transgenic lines in which this form of MEI-S332-GFP was expressed from the endogenous promoter at levels comparable to wild type (Figure S2). This protein was localized in spermatocytes lacking wild-type MEI-S332. The results contrasted with those obtained in cell culture in that the protein was delocalized from centromeres at the metaphase II/anaphase II transition (Figure 4). Nondisjunction assays were used to quantify the function of MEI-S332^T331-A^ in chromosome segregation. This mutant protein also restored sister-chromatid segregation to *mei-S332* null mutants (Table 2).

**Table 2:** Sex chromosome nondisjunction in males transheterozygous for *mei-S332* null alleles with transgenic *mei-S332^T331^* phosphomutants*^a^*

	Regular Sperm		Exceptional Sperm				Total Progeny	Total Exceptional Progeny (%)
	*Y (Y)*	*X*	Nullo-*XY* (%)	*XX* (%)	*XY(Y)* (%)	*XXY(Y)* (%)		
*mei-S332^7^ /mei-S332^4^*; *P{w^+^*, *mei-S332^T331-A^-GFP} line 1a*	767	829	1 (0.06%)	1 (0.06%)	5 (0.31%)	2 (0.12%)	1605	9 (0.56%)
*mei-S332^7^ /mei-S332^4^*; *P{w^+^*, *mei-S332^T331-A^-GFP} line 3a*	451	477	3 (0.32%)	0 (0%)	0 (0%)	0 (0%)	931	3 (0.32%)
*mei-S332^7^ /mei-S332^4^*; *P{w^+^*, *mei-S332^T331-A^-GFP} line 6a*	223	202	3 (0.70%)	0 (0%)	2 (0.46%)	0 (0%)	430	5 (1.16%)
*mei-S332^7^ /mei-S332^4^*; *P{w^+^*, *mei-S332^T331-A^-GFP} line 8b*	478	515	1 (0.01%)	1 (0.01%)	1 (0.1%)	0 (0%)	996	3 (0.30%)
*mei-S332^7^ /mei-S332^4^*; *P{w^+^*, *mei-S332^T331-D^-GFP} line 3a*	391	517	9 (0.97%)	0 (0%)	2 (0.22%)	0 (0%)	919	11 (1.20%)
*mei-S332^7^ /mei-S332^4^*; *P{w^+^*, *mei-S332^T331-D^-GFP} line 10b*	755	982	9 (0.51%)	0 (0%)	2 (0.11%)	0 (0%)	1748	11 (0.63%)

a See Table 1 for wild type, *meiS322* mutant controls, and wild-type transgene controls.

**Figure 5:**
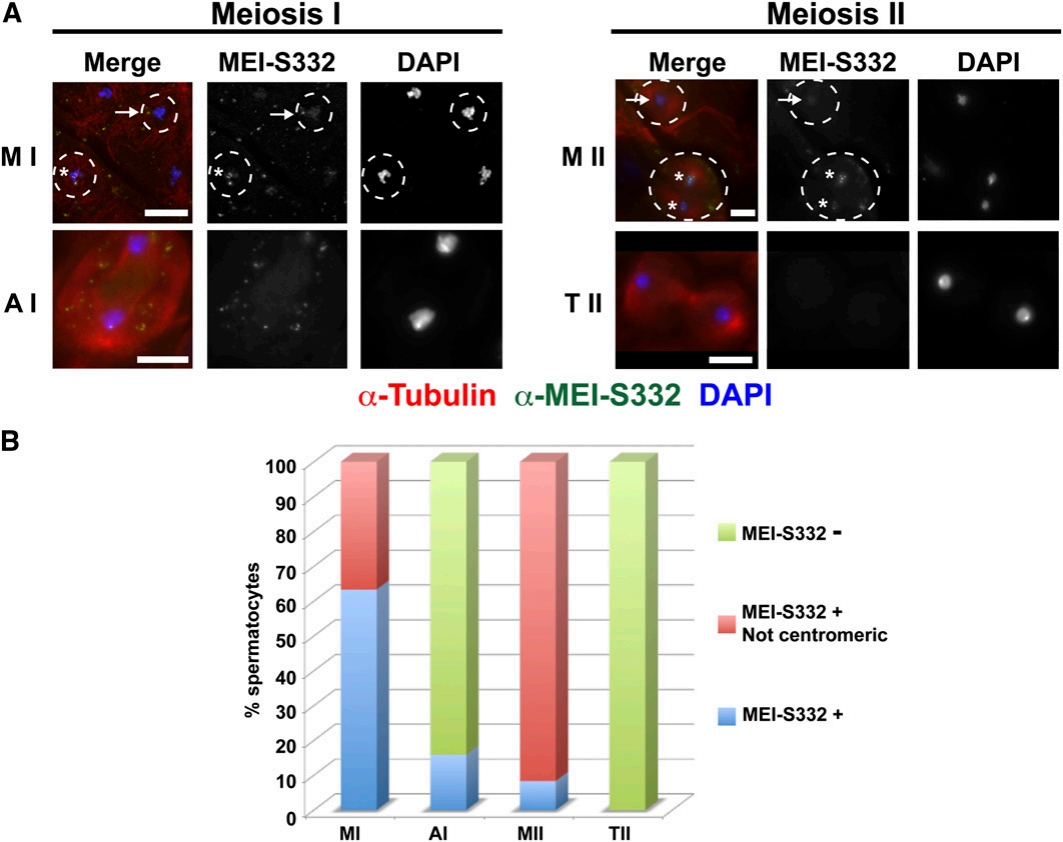
Localization of a MEI-S332 phosphorylation mimic in a POLO binding site (MEI-S332^T331-D^) during male meiosis. (A) Localization of MEI-S332^T331-D^ in meiosis I and II. Labels and scale bars as in Figure 1. The dotted lines highlight individual metaphase I or II spermatocytes, except two metaphase II spermatocytes are included in the large, bottom circle of the metaphase II panel. The arrow in the metaphase I panel shows a spermatocyte with MEI-S332^T331-D^ coating the chromosome arms, whereas the asterisk shows localization specifically to the centromeres. An early anaphase I spermatocyte is shown. All three metaphase II figures shown have MEI-S332^T331D^ all along the chromosomes (arrow in the top nucleus and asterisks in bottom circle containing two metaphase II figures). Puncta of MEI-S332-GFP are present in the cytoplasm of the meiosis I spermatocytes. (B) Quantification of MEI-S332^T331-D^ localization in metaphase I (n = 30), anaphase I (n = 63), metaphase II (n = 24), and telophase II (n = 457). Blue indicates MEI-S332 present solely at centromeres, red shows localization along the chromosome arms, and green indicates no detectable MEI-S332.

A phosphomimetic version of the Polo box-binding domain was made in which T331 was replaced with aspartic acid (T331-D). *In vitro* binding studies with Polo (see File S1) showed that this form of MEI-S332 exhibited enhanced binding to Polo (Figure S3). The behavior of MEI-S332-GFP^T331-D^ was examined in transgenic lines as for the other MEI-S332 phosphomutants (Figure 5). In metaphase I, the protein localized to centromeres in 60% of spermatocytes, but in the remaining spermatocytes, weak arm localization was present. The majority of anaphase I cells lacked detectable MEI-S332 on the chromosomes. Nearly all metaphase II cells showed weak staining for MEI-S332 along the chromosomes. Thus, the T331-D mutation reduces centromere localization of MEI-S332, instead there are low levels of protein binding along the chromosome arms.

Surprisingly, despite the reduction of MEI-S332^T331-D^ at the centromere, chromosomes segregated accurately (Table 2). This form of the protein restored proper segregation of the sex chromosomes to *mei-S332* null mutants. These genetic tests reveal that low, but detectable, levels of MEI-S332 at the centromere can protect cohesion.

### Function of the mutant protein forms in female meiosis

In Drosophila oocytes, as in vertebrate oocytes, meiosis arrests to permit oocyte differentiation. This necessitates retention of sister-chromatid cohesion for prolonged periods. We tested the function of the four phosphomutant forms of MEI-S332 in female meiosis by using nondisjunction tests to monitor segregation of the X chromosomes (Table 3 and Table 4). The S124,5,6-A phosphoresistant and S124,5,6-D phosphomimetic forms of MEI-S332 rescued the null *mei-S332* mutants at levels significantly the same as wild type, as did the T331-D phosphomimic. For the two lines of the Polo phosphomutant transgene lines in which more than 1000 progeny were scored, one showed full rescue. The other, T331-A #1a, gave slight nondisjunction that was significantly different from the wild-type transgene, despite the protein being expressed at normal levels (Figure S2). We conclude that as in males, the phosphomutant forms of MEI-S332 are capable of ensuring accurate chromosome segregation in female meiosis.

**Table 3:** Sex chromosome nondisjunction in females transheterozygous for *mei-S332* null alleles with transgenic *mei-S332^S124,5,6^* phosphomutants

	Regular Ova		Exceptional Ova		Total Progeny	Total Adjusted Progeny	Total Adjusted Exceptional Progeny (%)
	*X*	*X*	Nullo-*XX* (%)	*XX* (%)			
*yw;Tft/SM6*	308	214	0 (0%)	0 (0%)	522	522	0 (0%)
*mei-S332^7^ /mei-S332^4^*	137	145	33 (8.1%)	29 (7.1%)	344	465	124 (26.7%)
*mei-S332^7^ /mei-S332^4^*; *P{w^+^*, *mei-S332^+^-GFP}*	424	513	2 (0.21%)	0 (0%)	939	943	4 (0.42%)
*mei-S332^7^ /mei-S332^4^*; *P{w^+^*, *mei-S332^S124,5,6-A^-GFP} line 3b*	587	665	1 (0.08%)	2 (0.16%)	1255	1361	6 (0.44%)
*mei-S332^7^ /mei-S332^4^*; *P{w^+^*, *mei-S332^S124,5,6-A^-GFP} line 5a*	107	136	3 (1.19%)	1 (0.4%)	247	255	8 (3.1%)
*mei-S332^7^ /mei-S332^4^*; *P{w^+^*, *mei-S332^S124,5,6-A^-GFP} line7a**^a^*	3	3	0 (0%)	1 (12.5%)	7	9	2 (22.2%)
*mei-S332^7^ /mei-S332^4^*; *P{w^+^*, *mei-S332^S124,5,6-D^-GFP} line 4a*	394	491	5 (0.56%)	2 (0.22%)	892	906	14 (1.5%)
*mei-S332^7^ /mei-S332^4^*; *P{w^+^*, *mei-S332^S124,5,6-D^-GFP} line 6a*	47	58	0 (0%)	0 (0%)	105	105	0 (0%)
*mei-S332^7^ /mei-S332^4^*; *P{w^+^*, *mei-S332^S124,5,6-D^-GFP} line 13a*	1399	1457	32 (1.08%)	16 (0.54%)	2904	3000	96 (3.2%)
*mei-S332^7^ /mei-S332^4^*; *P{w^+^*, *mei-S332^S124,5,6-D^-GFP} line17c*	294	323	3 (0.48%)	1 (0.16%)	621	629	8 (1.3%)

a This line shows no transgenic protein expression by immunoblot (see Figure S2).

**Table 4:** Sex chromosome nondisjunction in females transheterozygous for *mei-S332* null alleles and with transgenic *mei-S332^T331^* phosphomutants*^a^*

	Regular Ova		Exceptional Ova		Total Progeny	Total Adjusted Progeny	Total Adjusted Exceptional Progeny (%)
	*X*	*X*	Nullo-*XX* (%)	*XX* (%)			
*mei-S332^7^ /mei-S332^4^*; *P{w^+^*, *mei-S332^T331-A^-GFP} line 1a*	607	655	6 (0.46%)	9 (0.7%)	1277	1307	30 (2.3%)
*mei-S332^7^ /mei-S332^4^*; *P{w^+^*, *mei-S332^T331-A^-GFP} line 3a*	575	603	2 (0.17%)	4 (0.34%)	1184	1196	12 (1.0%)
*mei-S332^7^ /mei-S332^4^*; *P{w^+^*, *mei-S332^T331-A^-GFP} line 6a*	15	30	0 (0%)	0 (0%)	45	45	0 (0%)
*mei-S332^7^ /mei-S332^4^*; *P{w^+^*, *mei-S332^T331-D^-GFP} line 3a*	176	189	0 (0%)	2 (0.54%)	367	371	4 (1.1%)
*mei-S332^7^ /mei-S332^4^*; *P{w^+^*, *mei-S332^T331-D^-GFP} line 10b*	442	485	1 (0.1%)	5 (0.53%)	933	945	12 (1.3%)

a See Table 3 for wild type, *mei-S332* mutant, and wild-type transgene controls.

## Discussion

The ability to combine cytology with quantitative analysis of chromosome segregation in Drosophila permitted us to time precisely the localization of MEI-S332 with respect to sister-chromatid cohesion and to exploit phosphomutant forms of MEI-S332 to define the relationship between MEI-S332 centromere localization and accurate chromosome segregation. This led to the key conclusions that inactivation of MEI-S332 to release centromere cohesion is distinct from delocalization of MEI-S332 and that MEI-S332 is not required on the centromere at detectable levels after anaphase I for proper meiosis II chromosome segregation in male meiosis.

We observed that wild-type MEI-S332 redistributes from the centromere to localize along the chromosome arms in anaphase I. This parallels the behavior of the INCENP subunit of the CPC in *Drosophila* spermatocytes (Resnick *et al.* 2006). Given the physical interaction between INCENP and MEI-S332, the dispersed chromosomal localization in anaphase I suggests that MEI-S332 may move with the CPC as it relocalizes; however, additional regulatory steps must unlink the colocalization between MEI-S332 and the CPC, as MEI-S332 does not move to the spindle midzone and is not present along the chromosomes in telophase I as is the CPC. It is striking that MEI-S332 reassociates with centromeres in meiosis II after having delocalized in late anaphase I, despite it apparently no longer being required for segregation. In mouse, the Sgol2 protein also delocalizes and then relocalizes to centromeres for meiosis II (Gomez *et al.* 2007; Parra *et al.* 2009). Proteins able to recruit MEI-S332 to centromeres, such as the CPC, may bring it along in meiosis II. In mouse, Sgol2 does localize to centromeres in meiosis II after the CPC is observed to bind. If the CPC is responsible for MEI-S332 centromere localization in meiosis II, the dependency must become uncoupled late in meiosis II, as INCENP is present on the chromosomes in telophase II, but MEI-S332 is not detectable (Resnick *et al.* 2006).

Analysis of the centromere localization of MEI-S332 confirms that in meiosis, as in mitosis, cohesion at the centromere can be released without delocalization of MEI-S332. The wild-type protein is detectable on the chromosomes of some anaphase II cells, and the clear segregation of sister chromatids indicates that despite this centromere cohesion has been released. This supports a mechanism to inactivate MEI-S332 distinct from delocalization. The tension model in which Sgo is pulled from the inner centromere to the kinetochore after stable bipolar microtubule attachment would provide one explanation. Although MEI-S332 was shown in colabeling studies to be present on the centromere rather than the kinetochore, these studies were done in prometaphase I (Lopez *et al.* 2000). Thus it is possible that as in the case of Sgol2 in mouse meiosis (Gomez *et al.* 2007; Parra *et al.* 2009), MEI-S332 may move to the kinetochore in metaphase II, physically removed and no longer able to protect cohesin. This could permit cleavage of cohesin without a requirement for delocalization of MEI-S332. Another mechanism of MEI-S332 inactivation cannot be excluded, however. Our localization studies do not map precisely the position of MEI-S332 with respect to the centromere, kinetochore, or centric heterochromatin in anaphase II cells, although they clearly show it is not present along the arms unless at very low levels.

The use of quantitative nondisjunction assays permitted us to analyze rigorously the function of the phosphomutant forms of MEI-S332 in male meiosis. Remarkably, all four protein forms function to ensure accurate meiotic segregation of homologs and sister chromatids. This is true even for those with reduced levels at the centromere in metaphase I, indicating that even lower amounts can nevertheless protect centromere cohesion at the metaphase I/anaphase I transition. A major insight from these studies is that meiosis II chromosome segregation occurs accurately in the absence of detectable MEI-S332 on the centromeres in male meiosis. Thus, in contrast to mammalian Sgol2, MEI-S332 is unlikely to have an essential role in chromosome congression, kinetochore biorientation, or inactivation of the spindle assembly checkpoint (Rattani *et al.* 2013). It also does not appear required for spindle assembly, as is Sgo2 in *Xenopus* (Rivera *et al.* 2012).

The localization properties of some of the phosphomutant forms of MEI-S332 differ from those observed in cell culture. The S124,5,6-A phosphomutant form of MEI-S332, which is resistant to Aurora B phosphorylation *in vitro* and in cell culture shows reduced centromere localization and localization along chromosome arms (Resnick *et al.* 2006), in meiosis also shows reduced centromere staining intensity in anaphase I but not arm localization. It is possible that there are other sites in MEI-S332 phosphorylated by Aurora B *in vivo*. In meiosis we did not observe retention of the MEI-S332^T331-A^ form resistant to Polo phosphorylation at anaphase II, as was observed in cell culture (Clarke *et al.* 2005). It is unclear why these phosphomutant protein forms show differences in localization in meiosis *vs.* cell culture, and it is possible that they affect other protein interactions rather than simply phosphorylation by Aurora B and Polo. Similarly, we do not yet know why the MEI-S332^S124,5,6-D^ phosphomimetic form is not detectable on chromosomes after anaphase I. The MEI-S332^T331-D^ mutant with enhanced Polo binding *in vitro* shows aberrant localization to the chromosome arms in metaphase I and II, a pattern that may reflect binding to Polo *in vivo*. It is possible that the three S124,5,6-A, S124,5,6-D, and T331-A phosphomutants all enhance delocalization of MEI-S332 from the centromere in meiosis II because centromere localization was not observed in the anaphase II cells. We are reluctant to make this conclusion, however, given that wild-type MEI-S332 was observed on less than 30% of anaphase II spermatocytes and small numbers of anaphase II cells were scored for the phosphomutants. Further delineation of the role of Aurora B and Polo phosphorylation on MEI-S332 in meiosis will require developing phosphor-specific antibodies.

MEI-S332 is the only Sgo family member identified in Drosophila, and thus one prediction might be it would contain all of the activities ascribed to Sgo proteins in mitosis and meiosis in other organisms. The ability of chromosomes to segregate accurately in meiosis II in the absence of detectable centromeric MEI-S332 indicates that in this division, as in mitosis, MEI-S332 is not essential. This does not exclude the possibility that it contributes to congression, kinetochore biorientation, or spindle assembly in a nonessential manner. In the future, live imaging of progression of spermatocytes through meiosis II with the phosphomutant forms of MEI-S332 may uncover such a role.

## Supplementary Material

Supporting Information
