# Supplementary material for: Regulation of Centromere Localization of the Drosophila Shugoshin MEI-S332 and Sister-Chromatid Cohesion in Meiosis
Source: G3 (Bethesda). 2014 Jul 31;4(10):1849–58. doi: 10.1534/g3.114.012823 (PMC4199692; doi:10.1534/g3.114.012823)
Supplement: Supporting Information [file supp_4_10_1849__index.html]

Regulation of Centromere Localization of the Drosophila Shugoshin MEI-S332 and Sister-Chromatid Cohesion in Meiosis — Supporting Information 

# Regulation of Centromere Localization of the *Drosophila* Shugoshin MEI-S332 and Sister-Chromatid Cohesion in Meiosis

## Supporting Information for Nogueira *et al.*, 2014

**Files in this Data Supplement:**

- Supporting Information - File S1 and Figures S1-S3 (PDF, 341 KB)
- File S1 - Supplementary Materials and Methods (PDF, 131 KB)
- Figure S1 - Specificity of the MEI-S332 antibody. (PDF, 142 KB)
- Figure S2 - Expression of MEI-S332 phosphomutant protein forms in transgenic flies. (PDF, 460 KB)
- Figure S3 - The MEI-S332T331-D mutant protein shows enhanced Polo binding. (PDF, 433 KB)
