# Supplementary material for: Regulation of Centromere Localization of the Drosophila Shugoshin MEI-S332 and Sister-Chromatid Cohesion in Meiosis
Source: G3 (Bethesda). 2014 Jul 31;4(10):1849–58. doi: 10.1534/g3.114.012823 (PMC4199692; doi:10.1534/g3.114.012823)
Supplement: Supporting Information [file supp_g3.114.012823_012823SI.pdf]

G3 · Genes | Genomes | Genetics

Regulation of Centromere Localization of the Drosophila Shugoshin MEI-S332 and Sister-Chromatid Cohesion in Meiosis

Cristina Nogueira*[1,2], Helena Kashevsky*[1], Belinda Pinto*, Astrid Clarke*[3], and Terry L. Orr-Weaver*[§4]

*Whitehead Institute and [§]Dept. of Biology, Massachusetts Institute of Technology, Cambridge, MA 02142

1 These authors contributed equally to this work.
2 Current Address: Centre for Genomic Regulation (CRG), Barcelona, Spain
3 Current Address: Gilead Pharmaceuticals, Branford, CT 06405
4 Corresponding author: weaver@wi.mit.edu; 617-258-5245

DOI: 10.1534/g3.114.012823

File S1

Supplementary Materials and Methods

Western Blots

50 ug of total protein from whole ovary extracts was loaded in each lane of a 10% Tris-HCL gel (Criterion, Bio-Rad). Blots were incubated with anti-MEI-S332 guinea pig antibody (at 1:10,000) overnight at 4°C. The signal was detected with HRP-conjugated antibodies using Pierce ECL Plus Western Blotting substrate.

GST Pull-Down Experiments

GST pull down was performed as previously described, except KC127 rather than S2 cells were used (CLARKE *et al.* 2005). KC167 cells were transfected with the constructs pPL17-GFP alone, pPL17-mei-S332^{WT}-GFP (LEE *et al.* 2004), pPL17-mei-S332$^{S234A+T331A}$ –GFP (CLARKE *et al.* 2005) and pPL17-mei-S332^{T331D} –GFP (CLARKE *et al.* 2005). Purified GST and GST-POLO Box Domain were a generous gift from Julie Welburn (Whitehead Institute). Following binding to glutathione beads, immunoblots were prepared and bound to a guinea pig anti-GFP antibody generously provided by Mary-Lou Pardue (Massachusetts Institute of Technology). After detecting the GFP-antibody by HRP-conjugated antibodies using Pierce ECL Plus Western Blotting substrate, the band intensities were quantified using the NIH Image J 1.31v software. The intensity of the bands in the GST pull-down experiment was normalized to the intensity of the input bands. The quantification of binding of the mutant forms of MEI-S332 was determined relative to the binding of wild-type MEI-S332-GFP.

Supplementary Literature Cited

Clarke, A. S., T. T. Tang, D. L. Ooi and T. L. Orr-Weaver, 2005 POLO kinase regulates the Drosophila centromere cohesion protein MEI-S332. Dev. Cell 8**:** 53-64.

Lee, J. Y., K. J. Dej, J. M. Lopez and T. L. Orr-Weaver, 2004 Control of centromere localization of the MEI-S332 cohesion protection protein. Curr. Biol. 14**:** 1277-1283.

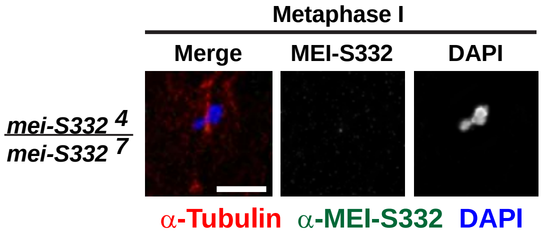

Figure S1 Specificity of the MEI-S332 antibody.

Shown is MEI-S332 staining of a metaphase I spermatocyte from a male transheterozygous for two *mei-S332* null alleles, *mei-S332*^{4}/*mei-S332*^{7}. We did not detect signal in the null mutant. Merged panels show MEI-S332 antibody staining in green, tubulin in red, and DAPI in blue. Split channels are shown for MEI-S332 and DAPI. Scale bar=10um.

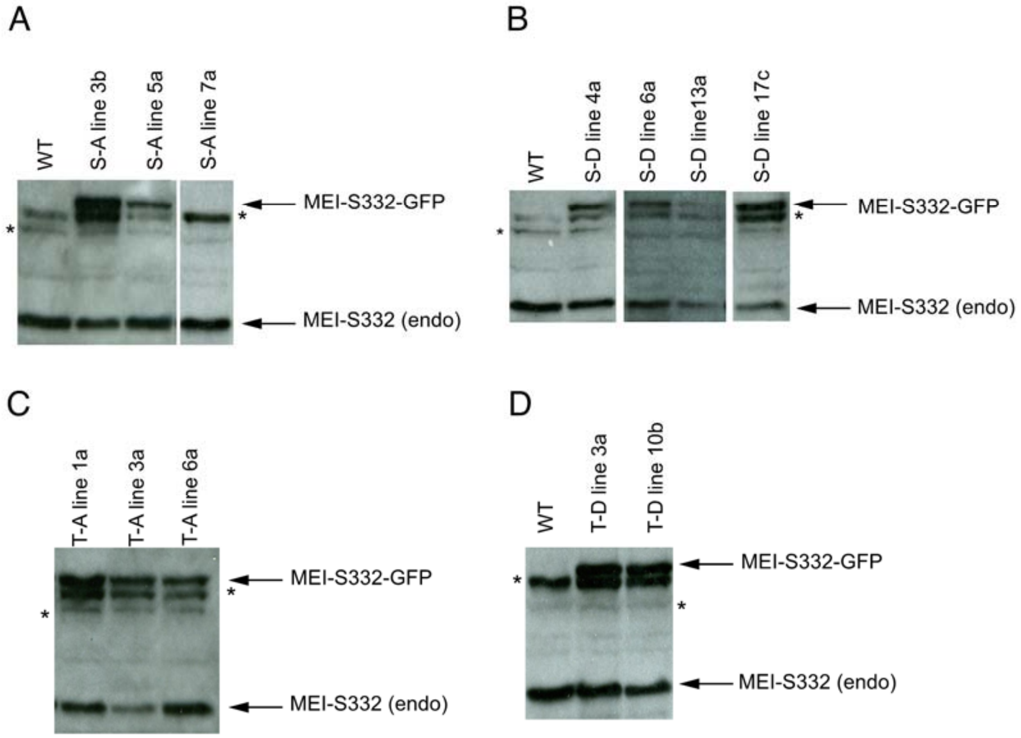

Figure S2 Expression of MEI-S332 phosphomutant protein forms in transgenic flies. Protein was extracted from whole ovaries, and immunoblots were probed with the MEI-S332 antibody. MEI-S332-GFP corresponds to the upper band (70kDa). Endogenous MEI-S332 corresponds to the lower band (50kDa) and serves as a loading control. WT are Oregon R flies not expressing MEI-S332 mutant proteins. Several unspecific bands are present, the most prominent marked by asterisks. (A) Three transgenic lines with MEI- S332$^{S124\text{-}126A}$-GFP. Line 7a does not express detectable levels of protein. (B) Four transgenic lines MEI- S332$^{S124\text{-}126D}$-GFP. (C) Three transgenic lines with MEI-S332$^{T331\text{-}A}$-GFP. (D) Two transgenic lines with MEI-S332$^{T331\text{-}D}$-GFP.

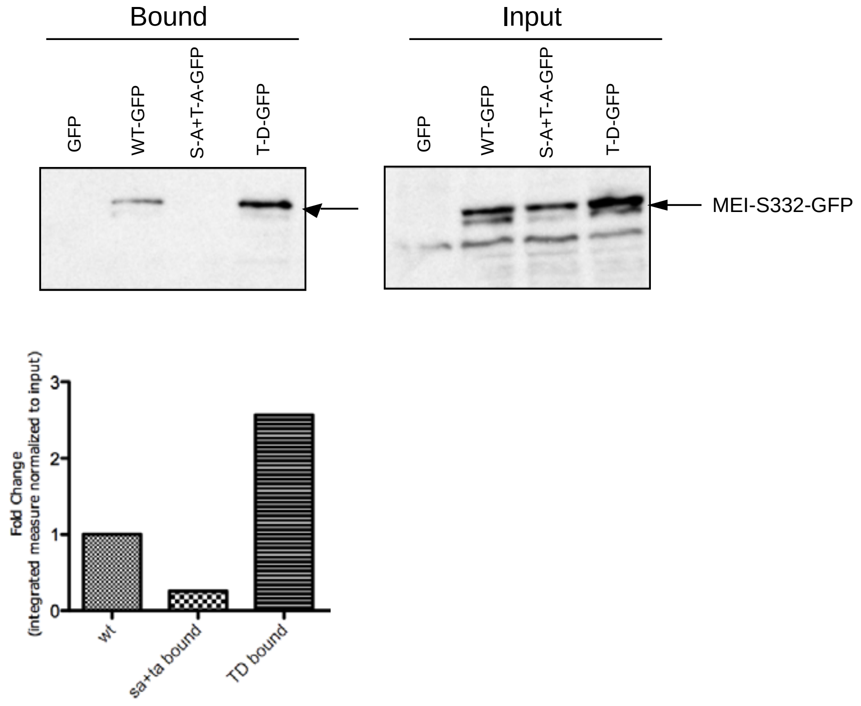

Figure S3 The MEI-S332$^{T331\text{-}D}$ mutant protein shows enhanced Polo binding. KC167 cells were transfected with genes encoding wild-type MEI-S332-GFP, MEI-S332-GFP with mutations in the predicted Polo Box Domain (PBD) binding domain of MEI-S332, or GFP alone as a control. MEI-S332$^{S234\text{-}A,\ T331\text{-}A}$ was previously shown to reduce binding to the PBD and is included solely as a control for comparison here (CLARKE *et al.* 2005). MEI-S332$^{T331\text{-}D}$ is a phosphomimic form predicted to enhance binding. Binding was measured by the ability of GST-Polo PBD expressed and purified from bacteria to pull down the MEI-S332-GFP proteins from KC167 extracts. Pull down was quantified by immunoblots with an antibody to GFP. The MEI-S332$^{S234\text{-}A,\ T331\text{-}A}$-GFP mutant protein shows a three-fold reduction of binding to GST-Polo PBD compared to wild-type MEI-S332-GFP. MEI-S332$^{T331\text{-}D}$-GFP shows enhanced binding (2.6 fold) compared to wild-type MEI-S332-GFP. GFP alone does not detectably bind GST-Polo PBD. Quantification of binding was normalized to input protein from the transfected KC167 cell extracts.
