# Supplementary material for: Regulation of Centromere Localization of the Drosophila Shugoshin MEI-S332 and Sister-Chromatid Cohesion in Meiosis
Source: G3 (Bethesda). 2014 Jul 31;4(10):1849–58. doi: 10.1534/g3.114.012823 (PMC4199692; doi:10.1534/g3.114.012823)
Supplement: Supporting Information [file supp_g3.114.012823_FileS1.pdf]

**File S1**

**Supplementary Materials and Methods**

**Western Blots**

50 ug of total protein from whole ovary extracts was loaded in each lane of a 10% Tris-HCL gel (Criterion, Bio-Rad). Blots were incubated with anti-MEI-S332 guinea pig antibody (at 1:10,000) overnight at $4^oC$. The signal was detected with HRP-conjugated antibodies using Pierce ECL Plus Western Blotting substrate.

**GST Pull-Down Experiments**

GST pull down was performed as previously described, except KC127 rather than S2 cells were used (CLARKE *et al.* 2005). KC167 cells were transfected with the constructs pPL17-GFP alone, pPL17-mei-$S332^{WT}$-GFP (LEE *et al.* 2004), pPL17-mei-$S332^{S234A+T331A}$ –GFP (CLARKE *et al.* 2005) and pPL17-mei-$S332^{T331D}$ –GFP (CLARKE *et al.* 2005). Purified GST and GST-POLO Box Domain were a generous gift from Julie Welburn (Whitehead Institute). Following binding to glutathione beads, immunoblots were prepared and bound to a guinea pig anti-GFP antibody generously provided by Mary-Lou Pardue (Massachusetts Institute of Technology). After detecting the GFP-antibody by HRP-conjugated antibodies using Pierce ECL Plus Western Blotting substrate, the band intensities were quantified using the NIH Image J 1.31v software. The intensity of the bands in the GST pull-down experiment was normalized to the intensity of the input bands. The quantification of binding of the mutant forms of MEI-S332 was determined relative to the binding of wild-type MEI-S332-GFP.

**Supplementary Literature Cited**

Clarke, A. S., T. T. Tang, D. L. Ooi and T. L. Orr-Weaver, 2005 POLO kinase regulates the Drosophila centromere cohesion protein MEI-S332. Dev. Cell 8**:** 53-64.

Lee, J. Y., K. J. Dej, J. M. Lopez and T. L. Orr-Weaver, 2004 Control of centromere localization of the MEI-S332 cohesion protection protein. Curr. Biol. 14**:** 1277-1283.
