# Supplementary material for: Regulation of Centromere Localization of the Drosophila Shugoshin MEI-S332 and Sister-Chromatid Cohesion in Meiosis
Source: G3 (Bethesda). 2014 Jul 31;4(10):1849–58. doi: 10.1534/g3.114.012823 (PMC4199692; doi:10.1534/g3.114.012823)
Supplement: Supporting Information [file supp_g3.114.012823_FigureS1.pdf]

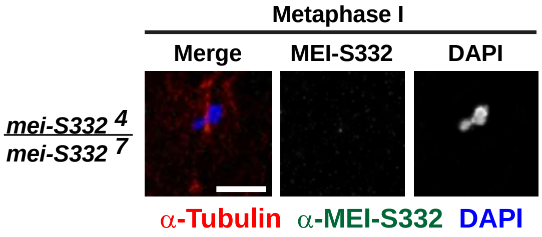

Figure S1 Specificity of the MEI-S332 antibody.

Shown is MEI-S332 staining of a metaphase I spermatocyte from a male transheterozygous for two *mei-S332* null alleles, *mei-S332*^{4}/*mei-S332*^{7}. We did not detect signal in the null mutant. Merged panels show MEI-S332 antibody staining in green, tubulin in red, and DAPI in blue. Split channels are shown for MEI-S332 and DAPI. Scale bar=10um.
