# Supplementary material for: Regulation of Centromere Localization of the Drosophila Shugoshin MEI-S332 and Sister-Chromatid Cohesion in Meiosis
Source: G3 (Bethesda). 2014 Jul 31;4(10):1849–58. doi: 10.1534/g3.114.012823 (PMC4199692; doi:10.1534/g3.114.012823)
Supplement: Supporting Information [file supp_g3.114.012823_FigureS2.pdf]

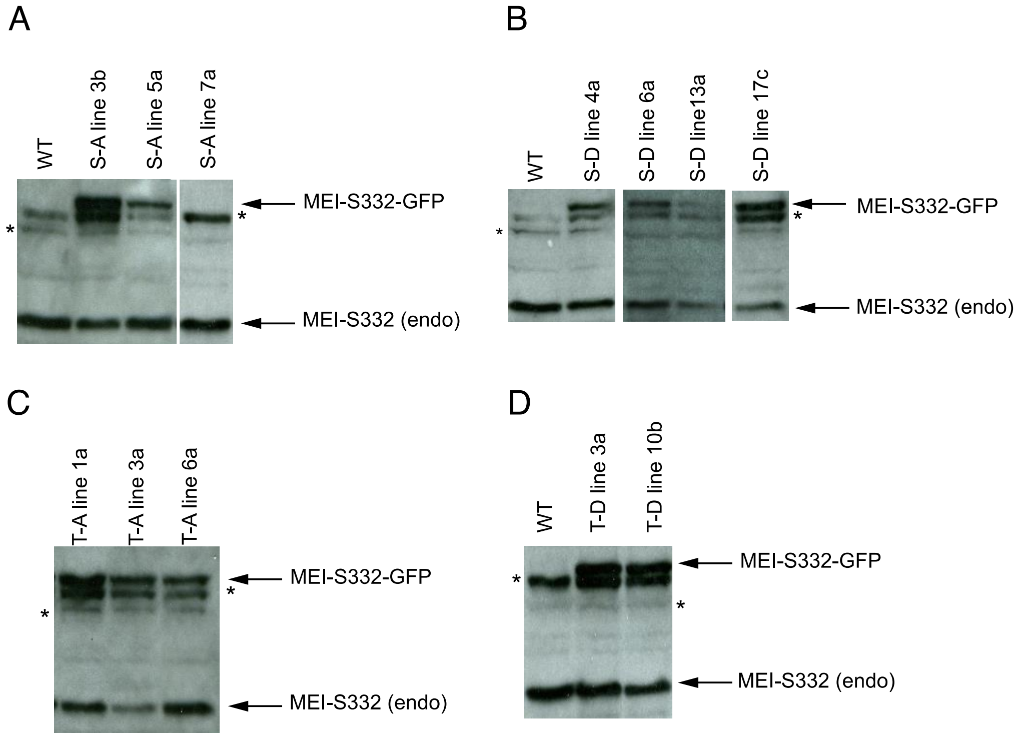

Figure S2 Expression of MEI-S332 phosphomutant protein forms in transgenic flies. Protein was extracted from whole ovaries, and immunoblots were probed with the MEI-S332 antibody. MEI-S332-GFP corresponds to the upper band (70kDa). Endogenous MEI-S332 corresponds to the lower band (50kDa) and serves as a loading control. WT are Oregon R flies not expressing MEI-S332 mutant proteins. Several unspecific bands are present, the most prominent marked by asterisks. (A) Three transgenic lines with MEI- S332$^{S124-126A}$-GFP. Line 7a does not express detectable levels of protein. (B) Four transgenic lines MEI- S332$^{S124-126D}$-GFP. (C) Three transgenic lines with MEI-S332^{T331-A}-GFP. (D) Two transgenic lines with MEI-S332^{T331-D}-GFP.
