# Supplementary material for: Regulation of Centromere Localization of the Drosophila Shugoshin MEI-S332 and Sister-Chromatid Cohesion in Meiosis
Source: G3 (Bethesda). 2014 Jul 31;4(10):1849–58. doi: 10.1534/g3.114.012823 (PMC4199692; doi:10.1534/g3.114.012823)
Supplement: Supporting Information [file supp_g3.114.012823_FigureS3.pdf]

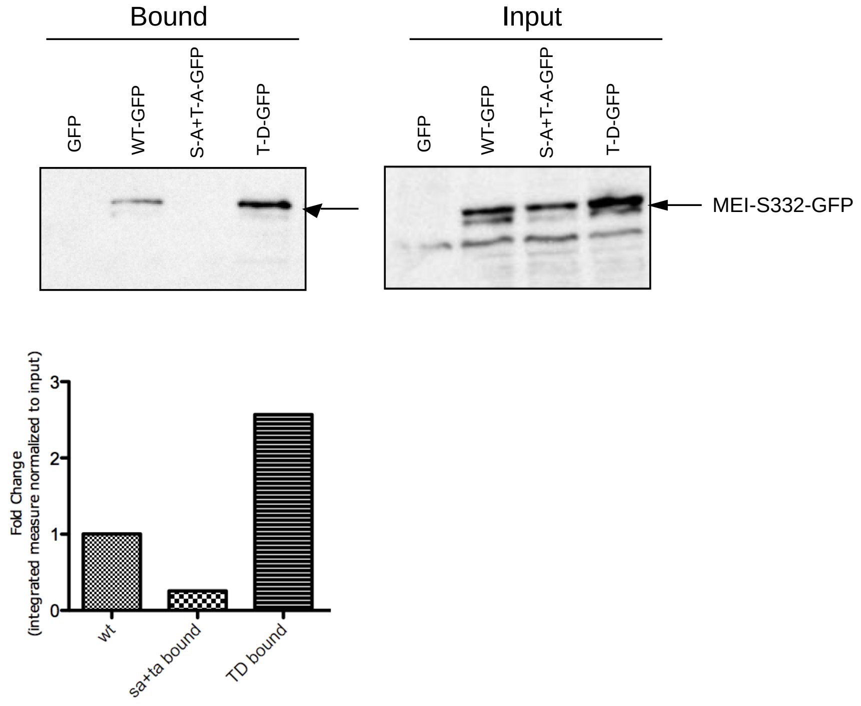

Figure S3 The MEI-S332$^{T331\text{-}D}$ mutant protein shows enhanced Polo binding. KC167 cells were transfected with genes encoding wild-type MEI-S332-GFP, MEI-S332-GFP with mutations in the predicted Polo Box Domain (PBD) binding domain of MEI-S332, or GFP alone as a control. MEI-S332$^{S234\text{-}A,\ T331\text{-}A}$ was previously shown to reduce binding to the PBD and is included solely as a control for comparison here (CLARKE *et al.* 2005). MEI-S332$^{T331\text{-}D}$ is a phosphomimic form predicted to enhance binding. Binding was measured by the ability of GST-Polo PBD expressed and purified from bacteria to pull down the MEI-S332-GFP proteins from KC167 extracts. Pull down was quantified by immunoblots with an antibody to GFP. The MEI-S332$^{S234\text{-}A,\ T331\text{-}A}$-GFP mutant protein shows a three-fold reduction of binding to GST-Polo PBD compared to wild-type MEI-S332-GFP. MEI-S332$^{T331\text{-}D}$-GFP shows enhanced binding (2.6 fold) compared to wild-type MEI-S332-GFP. GFP alone does not detectably bind GST-Polo PBD. Quantification of binding was normalized to input protein from the transfected KC167 cell extracts.
